# Immunohistochemical identification of *Propionibacterium acnes* in granuloma and inflammatory cells of myocardial tissues obtained from cardiac sarcoidosis patients

**DOI:** 10.1371/journal.pone.0179980

**Published:** 2017-07-07

**Authors:** Naoya Asakawa, Keisuke Uchida, Mamoru Sakakibara, Kazunori Omote, Keiji Noguchi, Yusuke Tokuda, Kiwamu Kamiya, Kanako C. Hatanaka, Yoshihiro Matsuno, Shiro Yamada, Kyoko Asakawa, Yuichiro Fukasawa, Toshiyuki Nagai, Toshihisa Anzai, Yoshihiko Ikeda, Hatsue Ishibashi-Ueda, Masanori Hirota, Makoto Orii, Takashi Akasaka, Kenta Uto, Yasushige Shingu, Yoshiro Matsui, Shin-ichiro Morimoto, Hiroyuki Tsutsui, Yoshinobu Eishi

**Affiliations:** 1 Department of Cardiovascular Medicine, Hokkaido University Graduate School of Medicine, Hokkaido, Japan; 2 Division of Surgical Pathology, Tokyo Medical and Dental University Hospital, Tokyo, Japan; 3 Department of Surgical Pathology, Hokkaido University Hospital, Hokkaido, Japan; 4 Department of Cardiovascular Medicine, Otaru-kyokai Hospital, Hokkaido, Japan; 5 Department of Cardiovascular Medicine, Sapporo City General Hospital, Hokkaido, Japan; 6 Department of Pathology, Sapporo City General Hospital, Hokkaido, Japan; 7 Department of Cardiovascular Medicine, National Cerebral and Cardiovascular Center, Osaka, Japan; 8 Department of Pathology, National Cerebral and Cardiovascular Center, Osaka, Japan; 9 Department of Cardiovascular Surgery, Machida Municipal Hospital, Tokyo, Japan; 10 Department of Cardiovascular Medicine, Wakayama Medical University, Wakayama, Japan; 11 Department of Pathology, Tokyo Women's Medical University, Tokyo, Japan; 12 Department of Cardiovascular and Thoracic Surgery, Hokkaido University Graduate School of Medicine, Hokkaido, Japan; 13 Department of Cardiology, Fujita Health University School of Medicine, Aichi, Japan; 14 Department of Cardiovascular Medicine, Kyusyu University, Fukuoka, Japan; 15 Department of Human Pathology, Graduate School and Faculty of Medicine, Tokyo Medical and Dental University, Tokyo, Japan; University of Ulster, UNITED KINGDOM

## Abstract

**Background:**

Although rare, cardiac sarcoidosis (CS) is potentially fatal. Early diagnosis and intervention are essential, but histopathologic diagnosis is limited. We aimed to detect *Propionibacterium acnes*, a commonly implicated etiologic agent of sarcoidosis, in myocardial tissues obtained from CS patients.

**Methods and results:**

We examined formalin-fixed paraffin-embedded myocardial tissues obtained by surgery or autopsy and endomyocardial biopsy from patients with CS (n = 26; CS-group), myocarditis (n = 15; M-group), or other cardiomyopathies (n = 39; CM-group) using immunohistochemistry (IHC) with a *P*. *acnes*-specific monoclonal antibody. We found granulomas in 16 (62%) CS-group samples. Massive (≥14 inflammatory cells) and minimal (<14 inflammatory cells) inflammatory foci, respectively, were detected in 16 (62%) and 11 (42%) of the CS-group samples, 10 (67%) and 10 (67%) of the M-group samples, and 1 (3%) and 18 (46%) of the CM-group samples. *P*. *acnes*-positive reactivity in granulomas, massive inflammatory foci, and minimal inflammatory foci were detected in 10 (63%), 10 (63%), and 8 (73%) of the CS-group samples, respectively, and in none of the M-group and CM-group samples.

**Conclusions:**

Frequent identification of *P*. *acnes* in sarcoid granulomas of originally aseptic myocardial tissues suggests that this indigenous bacterium causes granuloma in many CS patients. IHC detection of *P*. *acnes* in massive or minimal inflammatory foci of myocardial biopsy samples without granulomas may be useful for differentiating sarcoidosis from myocarditis or other cardiomyopathies.

## Introduction

Sarcoidosis, a systemic disease, is characterized by the presence of noncaseating epithelioid cell granulomas in multiple organs, including the heart, lung, skin, eyes, and central nervous system. Sarcoidosis is generally a benign disease, but cardiac involvement leading to functional abnormalities is an independent predictor for a poor prognosis [1]. Cardiac involvement is a major cause of sarcoid-related death [2], and affects 25% of patients with systemic sarcoidosis [3]. Corticosteroid therapy, a standard treatment for cardiac sarcoidosis (CS), improves the survival rate of CS patients by preventing left ventricular (LV) remodelling induced by repeated granulomatous inflammation and post-inflammatory fibrosis [4]. This powerful standard treatment for sarcoidosis, however, is only effective for CS patients with a preserved LV ejection fraction, and not those with advanced LV dysfunction [5]. Therefore, a definite CS diagnosis and rapid initiation of corticosteroid therapy are essential for improving the prognosis of CS patients. At present, however, the diagnosis of CS is challenging due to the variability of the clinical manifestations as well as the poor sensitivity of endomyocardial biopsy (EMB) caused by frequent sampling errors [6, 7].

Although the etiology of sarcoidosis remains undetermined, mycobacterial and propionibacterial organisms are the most commonly implicated etiologic agents [8]. To date, *Propionibacterium acnes* (*P*. *acnes*) is the only microorganism to be isolated from sarcoid lesions [9]. This indigenous bacterium was isolated from lymph node biopsy samples in 78% of sarcoidosis patients and 20% of non-sarcoidosis patients in Japan [10]. Recently, Negi et al. developed a novel monoclonal antibody specific to *P*. *acnes* (anti-*P*. *acnes* antibody) that reacts with cell membrane-bound lipoteichoic acid [11]. Immunohistochemistry (IHC) using the novel anti-*P*. *acnes* antibody revealed *P*. *acnes*-positive reactivity in sarcoid granuloma tissue samples from 74% of lungs and 88% of lymph nodes from patients with sarcoidosis, whereas no *P*. *acnes*-positive reactivity was detected in non-sarcoid granulomas of these organs from patients with tuberculosis or sarcoid reaction.

In the present study, we performed IHC with the *P*. *acnes*-specific monoclonal antibody to detect *P*. *acnes* in myocardial tissues from CS patients to evaluate the possible etiologic link of this bacterium with CS, and to estimate the feasibility of IHC analysis of *P*. *acnes* in myocardial biopsy samples to distinguish between CS and other cardiomyopathies.

## Methods

### Study samples

This study was designed as a retrospective multi-centre observational study. We collected 107 samples (61 EMB, 27 autopsy, and 19 surgical samples) from patients clinically diagnosed with CS (n = 39; CS-group), myocarditis (n = 17; M-group), or other cardiomyopathies (n = 51; CM-group) between January 1994 and April 2015 in seven Japanese Medical hospitals (Hokkaido University Hospital, National Cerebral and Cardiovascular Centre, Wakayama Medical University Hospital, Fujita Health University Hospital, Tokyo Women’s Medical University Hospital, Hayama Heart Centre, and Sapporo City General Hospital). We collected the EMB samples from the right ventricular septum. For the surgical samples, we punched a hole in the LV myocardial tissue to implant a left ventricular assist device or resected during left ventriculoplasty. The CS-group samples were collected from patients with histologically-proven myocardial sarcoidosis. All CS-group samples included one or more granulomas in the original histologic sections prepared for pathologic diagnosis. A definite diagnosis of CS was based on the presence of both myocardial granulomas and cardiac manifestation with or without extra-cardiac sarcoidosis involvement of at least one organ, according to the 2006 revised version of the Japanese Ministry of Health and Welfare guidelines [12]. CS patients with a clinical diagnosis but no histologic evidence were not included in the study. Myocarditis was diagnosed based on clinical manifestation, time-course, and histologic findings according to the ‘Guidelines for Diagnosis and Treatment of Myocarditis’ in Japanese Circulation Society Guidelines 2009 [13]. Histologic findings of myocarditis included inflammatory cell infiltration in the myocardium and the presence of adjacent necrotic and/or degenerated myocytes that were not typical of the ischaemic damage associated with coronary artery disease. Other cardiomyopathies, including dilated cardiomyopathy (DCM) and hypertrophic cardiomyopathy, were diagnosed by excluding CS and myocarditis, according to 2016 ESC Guidelines for the Diagnosis and Treatment of Acute and Chronic Heart Failure [14]. We excluded 27 samples with unexpected presence of non-specific background staining during IHC, and successful IHC results were obtained in tissue samples from 26 patients in the CS-group, 15 in the M-group, and 39 in the CM-group. Demographic and clinical data, including underlying heart disease, were obtained for all patients. For patients with EMB samples, we collected detailed information on the CS diagnosis. The ethics committee of Hokkaido University Hospital approved the study protocol, which was performed in accordance with the tenets of the Declaration of Helsinki. In the present study, individual patient consent was not required, but patients were informed of entry into the registry and allowed to opt out.

### Immunohistochemistry

Formalin-fixed paraffin-embedded myocardial tissues were obtained by EMB, or at cardiac surgery and autopsy. Serial paraffin sections on silane-coated slides (New Silane II, Muto Pure Chemicals, Tokyo, Japan) were stained with haematoxylin-eosin (HE) and immunostained with the anti-*P*. *acnes* antibody [11] and the anti-mycobacterial antibody [15] as a control. The IHC procedures were similar to those described previously [11]. Briefly, following deparaffinisation and rehydration, sections were microwaved at 97°C for 40 min (Microwave Processor H2850; Energy Beam Sciences, East Granby, CT, USA) in 10 mmol/l citrate buffer (pH 6.0) and then immersed for 10 min in 3% hydrogen peroxide in methanol. Initial incubation of the sections with normal horse serum (Vectastain Universal Elite ABC Kit; Vector Laboratories, Burlingame, CA, USA) was followed by a second overnight incubation at room temperature with the appropriately diluted antibody in a humidified chamber. The sections were then incubated at room temperature for 30 min with biotinylated secondary antibody, followed by another 30-min incubation at room temperature with streptavidin–peroxidase complex (Vectastain Universal Elite ABC Kit). Between each step, the sections were washed with phosphate-buffered saline containing 0.5% Tween-20. The peroxidase substrate diaminobenzidine (Histofine Simplestain DAB Solution; Nichirei Bioscience, Tokyo, Japan) was used to develop the signal as a brown reaction product, and all sections were counterstained with Mayer’s haematoxylin. IHC results for each sample were defined as positive when one or more small round bodies were detected within any of the granulomas or in any inflammatory cell infiltration foci, classified as either massive or minimal inflammatory foci. Massive inflammatory foci indicate clear inflammatory cell infiltration (diffuse, focal, or confluent) comprising ≥ 14 lymphocytes and macrophages/mm^2^ in the myocardium, consistent with the criteria that define myocarditis [13]. Minimal inflammatory foci, considered as non-specific findings, indicate ill-defined inflammatory cell infiltration (mostly focal) other than massive inflammatory foci [16]. Seven samples of CS-group, 5 samples of M-group, and 1 sample of CM-group contained both massive and minimum inflammatory foci. Therefore, we analyzed each of these lesions individually. The frequency of samples or lesions with *P*. *acnes*-positive reactivity by IHC in granulomas and massive or minimal inflammatory foci was compared between the CS-, M-, and CM-group samples and between the surgical/autopsy and EMB samples.

### Statistical analysis

Continuous variables are expressed as the mean ± standard deviation, and compared with a one-factor repeated-measures analysis of variance. Post-hoc comparisons were performed using Tukey’s Honestly Significant Difference test. Categorical variables are expressed as frequencies with percentages, and were compared using Fisher’s exact test followed by the Holm correction. A P value of less than 0.05 was considered statistically significant. Statistical analyses were performed using JMP 12 (SAS Institute, Cary, NC, USA).

## Results

### Patient characteristics

The patient demographics and clinical characteristics are summarized in Table 1. We examined 19 surgical, 27 autopsy, and 34 EMB samples. Mean patient age was significantly younger in the M-group than in the CS- and CM-groups (P’s < 0.001). The percentage of females was significantly higher in the CS-group than in the M- and CM-groups (P’s < 0.001). Detailed clinical profiles of patients from whom EMB samples were obtained are shown in Table 2. Similarly, the M-group patients were younger than those in the CS- and CM-groups (P = 0.006 and P = 0.002, respectively). Heart rate was lower in the CS-group patients than in the M-group patients (P = 0.003). Plasma angiotensin-1-converting enzyme levels were higher in the CS-group patients than in the M- and CM-group patients (P = 0.017 and P < 0.001, respectively). Serum troponin T was higher in the M-group patients than in the CM-group patients (P = 0.031). Extra-cardiac sarcoidosis, including that in the lung, eye, and skin, was detected in six (75%) of the CS-group patients. The clinical manifestations were isolated to the heart in two (25%) patients.

**Table 1:** Patient characteristics.

	CS-group (N = 26)	M-group (N = 15)	CM-group (N = 39)
Type of sample			
Surgical, n (%)	4 (15)	5 (33)	10 (26)
Autopsy, n (%)	14 (54)	3 (20)	10 (26)
EMB, n (%)	8 (31)	7 (47)	19 (49)
Age (years), range	27–92	15–79	18–85
Age (years), mean ± SD	61.5±8.3	40.3±19.5*^‡^	58.6±13.8
Female, n (%)	20 (77)^†^^‡^	2 (13)	10 (26)
Underlying heart disease			
Cardiac sarcoidosis, n (%)	26 (100)	-	-
Myocarditis	-	15 (100)	-
DCM, n (%)	-	-	25 (64)
HCM, n (%)	-	-	8 (21)
Others, n (%)	-	-	7 (18)

Continuous variables are expressed as mean ± SD and categorical variables are expressed as number (%) of patients.

*P<0.001 vs. CS-group;

^†^ P<0.001 vs. M-group;

^‡^P<0.001 vs. CM-group;

EMB, endomyocardial biopsy; DCM, dilated heart disease; HCM, hypertrophic heart disease.

**Table 2:** Clinical profiles of the patients providing EMB samples.

	CS-group (N = 8)	M-group (N = 7)	CM-group (N = 19)
Age (years), range	48–85	15–79	40–85
Age (years), mean ± SD	63.8±10.6	38.9±22.1*^†^	62.2±11.9
Female, n (%)	5 (63)	0 (0)	5 (26)
HR (beats/min)	63.7±16.7^‡^	91.4±20.3	82.9±20.0
SBP (mmHg)	108.0±17.1	104.4±21.3	116.3±24.7
LVEF (%)	37.5±14.5	28.6±20.2	38.9±19.5
Basal thinning of IVS, n (%)	3 (38) ^‡^	0 (0)	0 (0)
Uptake Ga scintigraphy or FDG-PET, n (%)	7/8 (88)	1/1 (100)	0/2 (0)
LGE-CMR, n (%)	3/3	1/3	13/14
Biochemistry			
Calcium (mEq/l)	9.3±0.4	8.7±0.3	9.1±0.2
BNP (pg/ml)	468.3±564.6	710.8±721.2	601.0±566.7
ACE (IU/L)	24.2±8.3^§^^∥^	12.0±5.3	9.3±6.5
Lysozyme (μg/mL)	7.8±1.3	8.0±2.4	9.0±2.4
sIL2-R (μg/mL)	394.0±130.0	-	-
Troponin T (ng/ml)	0.03±0.01	6.3±9.1^¶^	0.02±0.03
AV block, n (%)	4 (50)	2 (29)	1 (5)
VT or VF	2 (25)	0 (0)	0 (0)
PPM/ICD/CRT, n (%)	5 (63)	0 (0)	4 (21)
Extra-cardiac sarcoidosis	6 (75)	-	-
Medications			
Corticosteroid, n (%)	6 (75)^∥^	2 (29)	0 (0)
ACE-Is / ARBs, n (%)	5 (63)	6 (86)	16 (84)
β-blockers, n (%)	6 (75)	3 (43)	17 (90)
Diuretics, n (%)	2 (25)	4 (57)	13 (68)

All values are expressed as the mean ± SD or number (%) of patients. P < 0.05 was considered statistically significant.

*P = 0.006 vs. CS-group;

^†^P = 0.002 vs. CM-group;

^‡^P = 0.003 vs. M-group;

^§^P = 0.017 vs. M-group;

^∥^P < 0.001 vs. CM-group;

^¶^P = 0.031 vs. CM-group;

EMB, endomyocardial biopsy; HR, heart rate; SBP, systolic blood pressure; LVEF, left ventricular ejection fraction; IVS, intraventricular septum; Ga, gadolinium; FDG-PET, 18F-fluorodeoxy glucose-positron emission tomography; LGE, late gadolinium enhancement; CMR, cardiac magnetic resonance; BNP, brain-type natriuretic peptide; ACE, angiotensin converting enzyme; IL2-R, interleukin-2 receptor; AV, atrio-ventricular; VT, ventricular tachycardia; VF, ventricular fibrillation; PPM, permanent pacemaker; ICD, implantable cardioverter defibrillator; CRT, cardiac resynchronization therapy; ACE-I, angiotensin converting enzyme inhibitor; ARB, angiotensin II receptor blocker.

### Histologic findings

**Fig 1:**
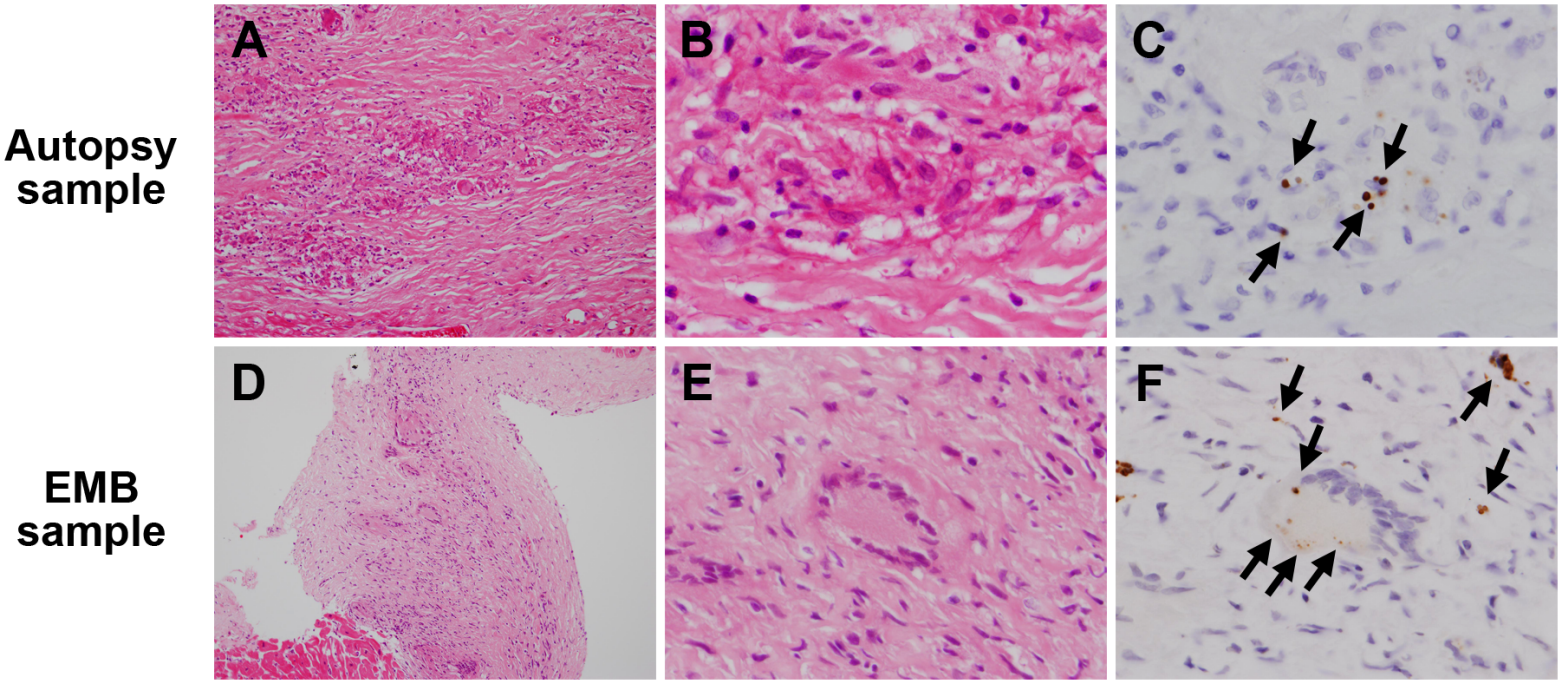
Representative samples with sarcoid granulomas. An autopsy sample (**A–C**) and an EMB sample (**D–F**) from patients with sarcoidosis were stained with haematoxylin and eosin and immunostained with anti-*P*. *acnes* antibody. Many sarcoid granulomas were observed at the lower magnification (**A, D**). Small round bodies indicated by the black arrows (**C, F**) were found in some of epithelioid cells and multinucleated giant cells of these sarcoid granulomas by immunohistochemistry with anti-*P*. *acnes* antibody. Original magnification; ×200 (left), ×1000 (middle and right).

**Fig 2:**
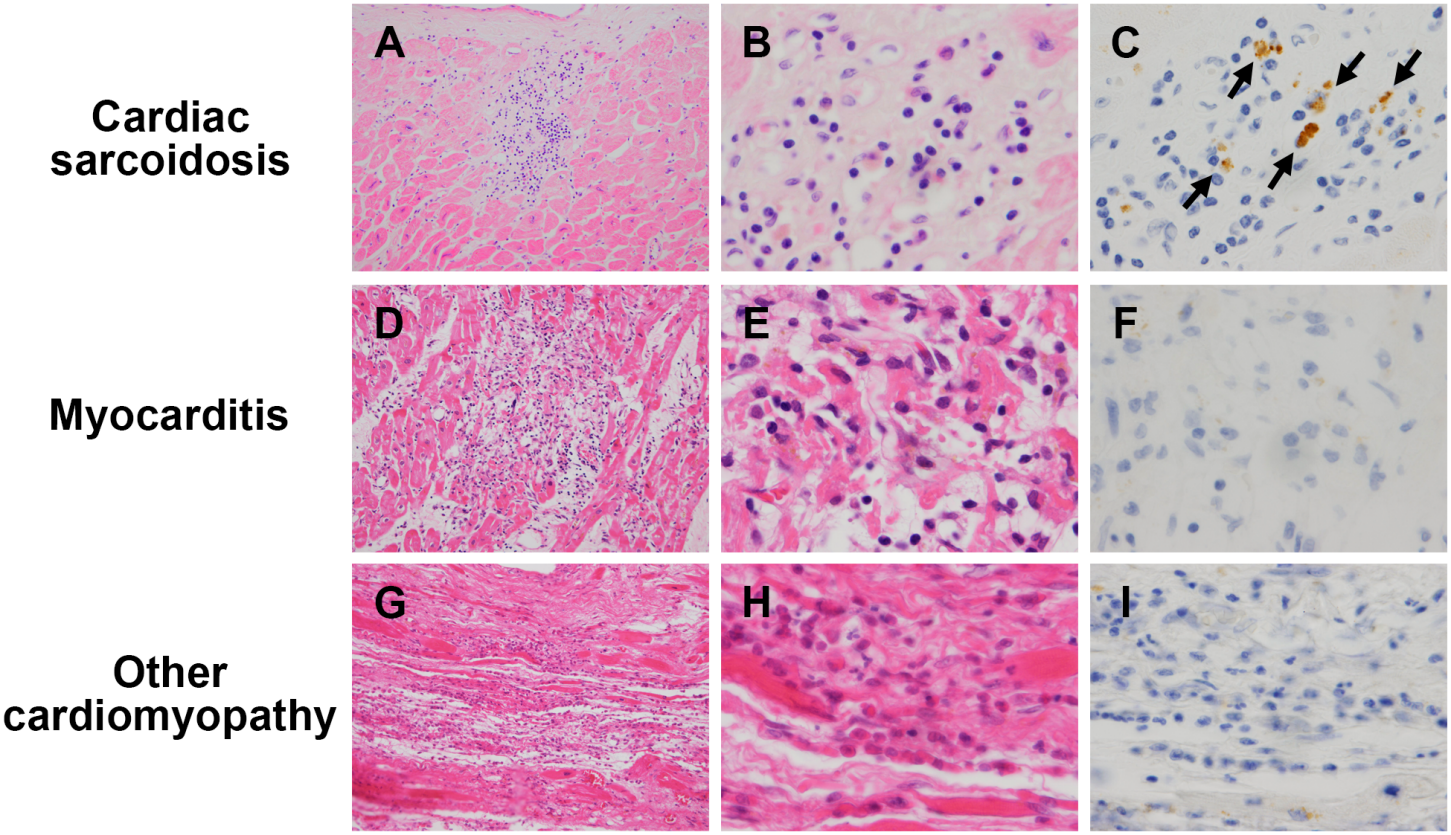
Representative samples with massive inflammatory foci. Specimens obtained from autopsy samples in patients with CS (**A–C**), myocarditis (**D–F**), or other cardiomyopathy (**G-I**) were stained by haematoxylin and eosin and immunostained with anti-*P*. *acnes* antibody. Massive inflammatory cell infiltration was observed in samples from patients with CS (**A, B**), myocarditis (**D, E**), or other cardiomyopathies (**G, H**). Positive *P*. *acnes* immunostaining in macrophages of these inflammatory foci was detected only in samples from patients with CS (**C**; black arrows), and not in samples from those with myocarditis (**F**) or other cardiomyopathies (**I**). Original magnification; ×200 (left), ×1000 (middle and right).

**Fig 3:**
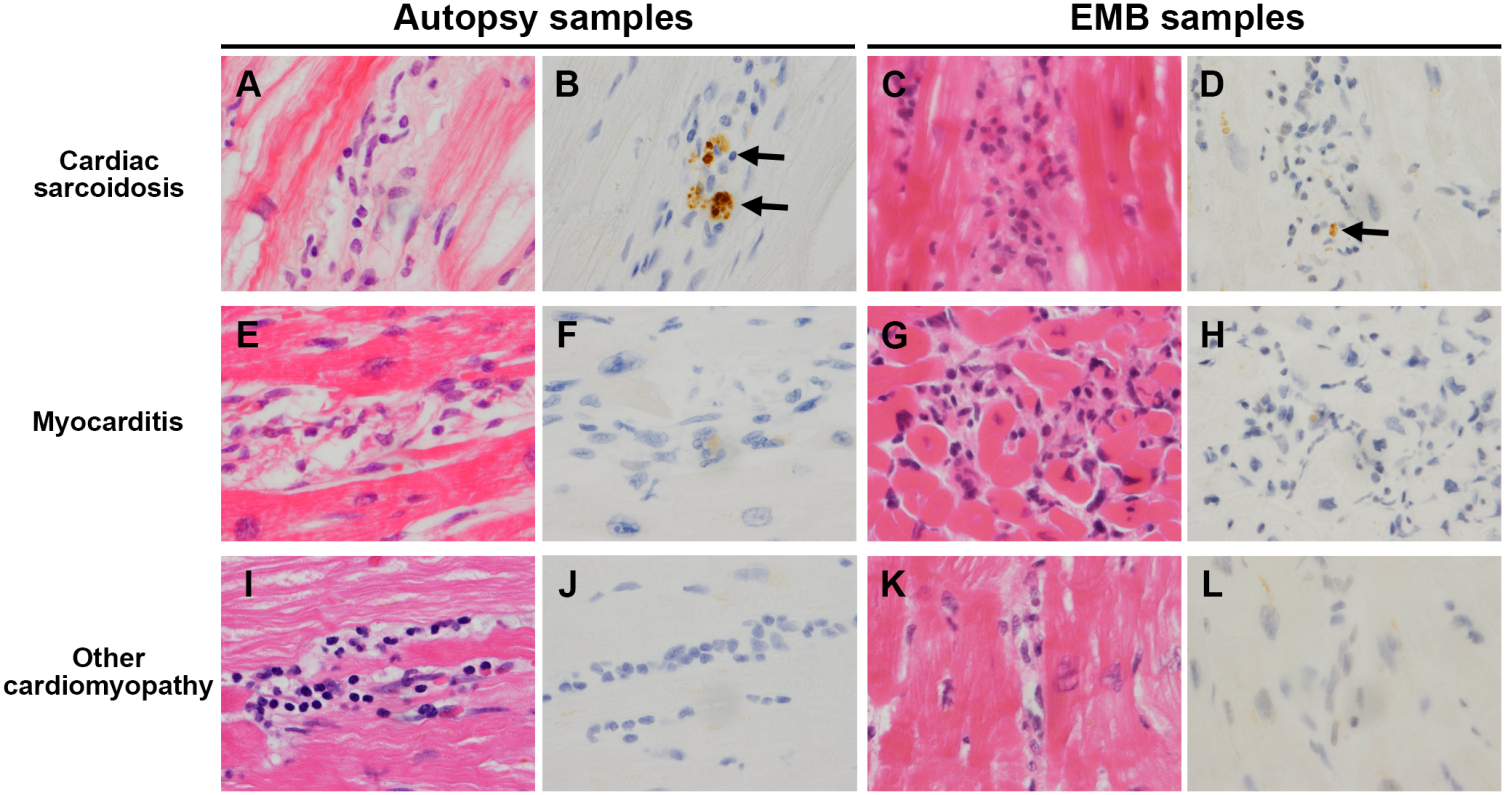
Representative samples with minimal inflammatory foci. Specimens obtained from autopsy samples and EMB samples in patients with CS (**A–D**), M (**E–H**), and CM (**I–L**) were stained by haematoxylin and eosin (**A, C, E, G, I,** and **K**) and immunostained with anti-*P*. *acnes* antibody (**B, D, F, H, J,** and **L**). Black arrows indicate positive *P*. *acnes*-immunostaining. Minimal inflammatory cell infiltration was observed in samples from all six patients. Even at the lowest inflammatory cell infiltration, positive *P*. *acnes*-immunostaining in macrophages of these inflammatory foci was detected in samples from patients with CS (**B, D**), but not in samples from those with M (**F, H**) and CM (**J, L**), regardless of the sample type. Original magnification; ×1000.

The number and frequency (%) of samples with granulomas or inflammatory cells (massive or minimal inflammatory foci) detected by HE, and the number and frequency (%) of such samples with *P*. *acnes* detected by IHC in each corresponding lesion are summarised for the surgical or autopsy samples and the EMB samples from each patient group (Table 3). Noncaseating epithelioid cell granulomas with or without multinucleated giant cells are shown in Fig 1. Granulomas were detected in 16 (62%) samples from the CS-group and in none of the samples from the M- and CM-groups. Massive inflammatory foci, as presented in Fig 2, were observed in 16 (62%) lesions from the CS-group and in 10 (67%) lesions from the M-group samples with no significant difference between them. Massive inflammatory foci were observed in only 1 (3%) lesion from the CM-group, significantly less frequently than in the CS- and M-group samples (P’s < 0.001). Minimal inflammatory foci, as shown in Fig 3, were also observed frequently in 11 (42%) samples from the CS-group, 10 (67%) samples from the M-group, and 18 (46%) samples from the CM-group, with no significant difference among groups. No granuloma was observed in the sections additionally prepared for the study in 10 (38%) of the CS-group samples, despite the fact that granulomas were identified in the initially prepared sections used for pathologic diagnosis. In the CS-group samples with no granuloma observed, 5 (50%) samples lacked any inflammatory lesions, including massive or minimal inflammatory foci. The frequency of granuloma and massive or minimal inflammatory foci was not significantly different between the surgical or autopsy and EMB samples.

**Table 3:** Summary of histologic and immunohistochemical findings.

	Number of samples	Number (%) of samples with granuloma (HE) and number (%) of the above samples with *P*. *acnes* detected in granulomas (IHC)		Number (%) of samples with massive inflammatory foci (HE) and number (%) of the above lesions with *P*. *acnes* detected therein (IHC)		Number (%) of samples with minimal inflammatory foci (HE) and number (%) of the above lesions with *P*. *acnes* detected therein (IHC)	
		HE	IHC	HE	IHC	HE	IHC
**CS-group**							
All samples	26	16 (62)	10 (63)	16 (62)*	10 (63)^§^	11 (42)	8 (73)^¶^*
Surgical/autopsy samples	18	10 (56)	5 (50)	10 (56)^†^	5 (50)	9 (50)	6 (67)**
EMB samples	8	6 (75)	4 (67)	6 (75)*	5 (83)^∥^	2 (25)	2 (100)^††^
**M-group**							
All samples	15	0	-	10 (67)*	0	10 (67)	0
Surgical/autopsy samples	8	0	-	6 (75)^†^	0	5 (63)	0
EMB samples	7	0	-	4 (57)^‡^	0	5 (71)	0
**CM-group**							
All samples	39	0	-	1 (3)	0	18 (46)	0
Surgical/autopsy samples	20	0	-	1 (5)	0	7 (35)	0
EMB samples	19	0	-	0	-	11 (58)	0

All values are expressed as the number (%) of patients.

*P < 0.001 vs. CM-group;

^†^P = 0.002 vs. CM-group;

^‡^P = 0.007 vs. CM-group;

^§^P = 0.003 vs. M-group;

^∥^P = 0.048 vs. M-group;

^t¶^P = 0.002 vs. M-group;

**P = 0.034 vs. CM-group;

^††^P = 0.038 vs. CM-group;

HE, haematoxylin-eosin; EMB, endomyocardial biopsy; IHC, immunohistochemistry.

#### Immunohistochemical findings

IHC with the anti-*P*. *acnes* antibody revealed one or a few small round bodies in some of sarcoid granuloma cells, including epithelioid cells and multinucleated giant cells (Fig 1). Such *P*. *acnes*-positive reactivity in granulomas was found in 10 (63%) of 16 CS-group samples with granuloma (Table 3). A few or many small round bodies were also detected in sarcoid inflammatory cells including massive (Fig 2) and minimal (Fig 3) inflammatory foci. *P*. *acnes*-positive reactivity in massive inflammatory foci was detected in 10 (63%) of the 16 CS-group samples and in none of the 10 M-group samples or 1 exceptional CM-group sample with massive inflammatory foci. The difference in the detection frequency between the CS-group and M-group samples was statistically significant (P = 0.003). *P*. *acnes*-positive reactivity in minimal inflammatory foci was detected in 8 (73%) of the 11 CS-group samples and in none of the 10 M-group and 18 CM-group samples with minimal inflammatory foci. The difference in the detection frequency between the CS-group and M-group or CM-group samples was statistically significant (P = 0.002 and P < 0.001), respectively. The frequency of *P*. *acnes*-positive reactivity in granulomas and massive or minimal inflammatory foci was not significantly different between the surgical or autopsy samples and the EMB samples, or between the CS-group patients with and without clinical characteristics, including extra-cardiac manifestations. Granulomas were not detected in the 10 (38%) of 26 CS-group samples. *P*. *acnes*-positive reactivity was detected within inflammatory foci even in 3 of 10 CS-group samples without granulomas. No positive reactivity was detected by the control IHC with the anti-mycobacterial antibody in any samples from the CS-group, M-group, and CM-group patients.

## Discussion

In this study, we identified *P*. *acnes* by IHC, for the first time, in myocardial tissues from CS patients. *P*. *acnes*-positive reactivity in granuloma cells was found in 63% of the CS-group samples with granulomas. *P*. *acnes*-positive reactivity was also found in massive and minimal inflammatory foci, in 63% and 73% of the CS-group samples, respectively, and in none of the M-group and CM-group samples with each corresponding lesion. The high frequency and specificity of the detection of the indigenous bacterium in sarcoid granuloma and inflammatory cells from originally aseptic myocardial tissues more strongly implicates its involvement in sarcoidosis etiology than earlier similar detection of *P*. *acnes* in sarcoid granulomas of the lungs and lymph nodes because the bacterium is thought to be commensal to these organs [17]. The results also suggest that IHC detection of *P*. *acnes* in massive or minimal inflammatory foci of myocardial biopsy samples without granulomas could be useful for differentiating sarcoidosis from myocarditis or other cardiomyopathies.

A definitive CS diagnosis usually requires a histologic examination of tissue samples obtained by EMB. A previous study demonstrated that a histologic CS diagnosis is limited to less than 30% of small EMB samples obtained from CS patients [6]; this is also the case for the hearts of CS patients obtained at autopsy [3]. We collected CS-group samples from histologically-proven CS patients with granulomas identified in the myocardial tissue sections originally prepared for pathologic diagnosis. Sarcoid granulomas were not found in 10 (38%) of the CS-group samples, but rather in the histologic sections that were additionally prepared for the present study. Similarly, routine pathologic diagnostic processes with no granuloma detected in the original sections but later found in the deep-cut sections of EMB samples sometimes occurred. This type of tissue-preparation error seems to be one of the problems, in addition to the more-common sampling errors, in CS diagnosis with EMB samples.

Granulomas in myocardial tissues, even when detected in surgical or autopsy samples and EMB samples, require a differential diagnosis between CS and other granulomatous diseases [18]. A minimum amount of focal and central eosinophilic necrosis in sarcoid granulomas may need to be differentiated from tuberculosis or other infectious diseases [19]. Similarly, giant cells that appear in giant cell myocarditis are sometimes difficult to distinguish from giant cells intermingled with sarcoid inflammatory cells [20–22]. In the CS-group samples with granulomas present, *P*. *acnes*-positive reactivity in the granulomas was found in 56% of the surgical or autopsy samples and 75% of the EMB samples with no significant difference between the large and small myocardial tissue samples. Negi et al. reported that no *P*. *acnes*-positive reactivity is found in non-sarcoid granulomas of lungs and lymph nodes from patients with tuberculosis or sarcoid reaction [11]. Therefore, IHC detection of *P*. *acnes* may be useful for distinguishing sarcoid granulomas from other non-sarcoidosis granulomas with a similar histologic appearance.

Additionally, minimal inflammatory cell infiltration in the myocardium is observed in some patients with DCM classified as inflammatory dilated cardiomyopathy (DCMI) [16, 23]. A small number of inflammatory cells are found in biopsy samples from patients with clinically suspected CS, but no granulomas. In such cases, a pathologic CS diagnosis cannot be made with only non-specific findings, and conventional heart failure therapy for idiopathic DCM is commonly initiated. It is therefore important to determine whether the inflammatory cell infiltration was caused by DCMI or CS. In the present study, *P*. *acnes*-positive reactivity was detected in 63% of the CS-group samples with massive inflammatory foci and in 73% of those with minimal inflammatory foci. Three autopsy samples from the CS-group with *P*. *acnes* detected in inflammatory foci contained only inflammatory lesions without granuloma in these histologic sections. These findings suggest that IHC with anti-*P*. *acnes* antibody may be useful for differentiating sarcoidosis from non-sarcoidosis myocarditis and other cardiomyopathies, especially when no granuloma is found but some inflammatory cells are observed in the myocardial biopsy samples.

*P*. *acnes*-positive reactivity was also observed in many samples with sarcoid granulomas and accompanying inflammatory foci (presumably a precursor lesion of granuloma formation caused by an identical pathogen). In general, granulomas are formed to sequester and degrade an invading agent. An epithelioid cell granuloma is the pathologic hallmark of sarcoidosis; thus, the sarcoid granuloma must contain, or have contained, an etiologic agent of sarcoidosis. According to these basic principles of granuloma formation, the high frequency and specificity of *P*. *acnes* detected in sarcoid granuloma and inflammatory cells in originally aseptic myocardial tissues suggest that this indigenous bacterium causes granuloma formation in many CS patients.

**Fig 4:**
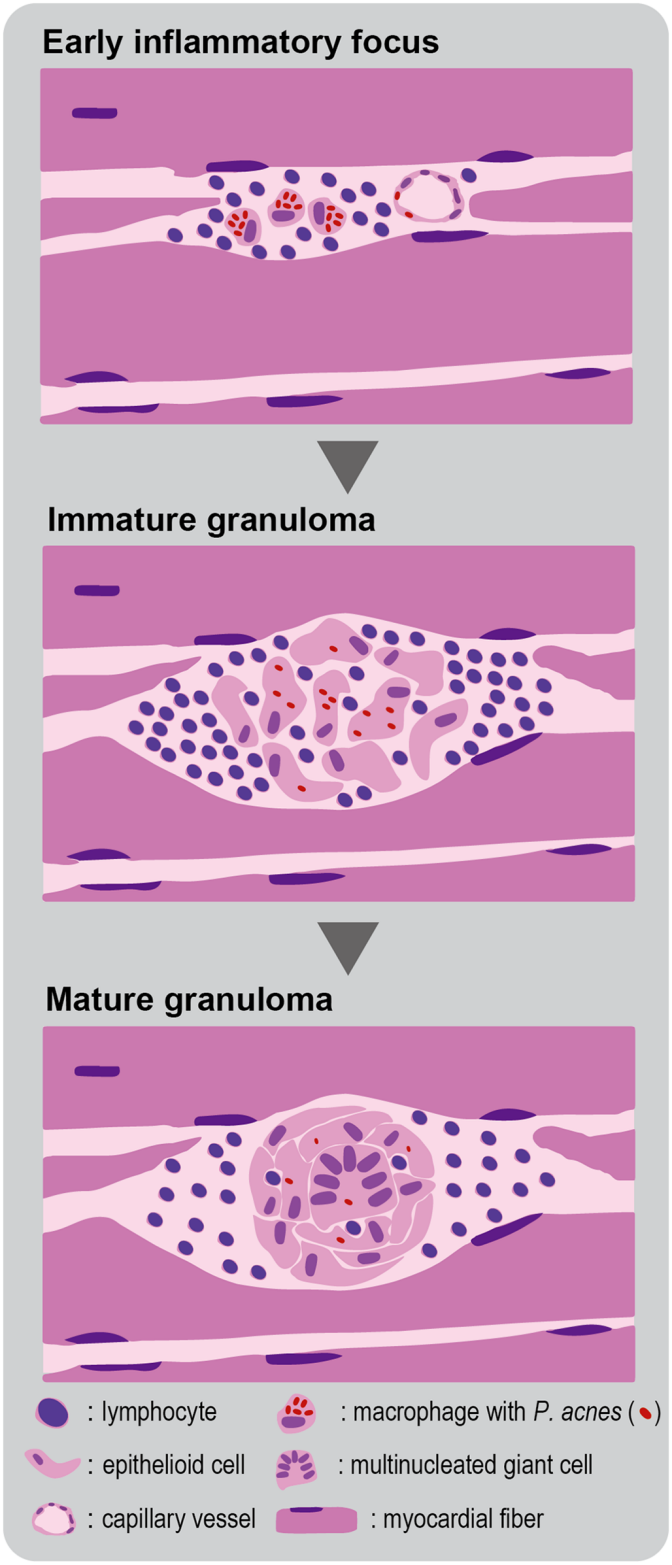
Schematic representation of granulomatous inflammation caused by *P*. *acnes*. Granulomas begin as small collections of lymphocytes and macrophages with intracellular *P*. *acnes* (early inflammatory foci) as observed in the minimal or massive inflammatory foci of the CS-group samples. Macrophages change to epithelioid cells and become organized into a cluster of cells (immature granuloma). Further progression results in ball-like clusters of cells and fusion of macrophages into giant cells with or without remaining intracellular *P*. *acnes* (mature granuloma).

A hypersensitive Th1 immune response to this commensal bacterium is thought to lead to granuloma formation in the susceptible subjects [24–26]. Based on the hypothesis that sarcoidosis is an allergic endogenous infection caused by *P*. *acnes* [27], *P*. *acnes* can cause latent infection primarily in the lungs and lung hilar lymph nodes and the secondarily in other systemic organs, such as the heart, eyes, and skin. The dormant form of *P*. *acnes* is activated endogenously under certain environmental conditions and proliferates at the site of latent infection. In patients with *P*. *acnes* hypersensitivity, granulomas are formed around macrophages with intracellular *P*. *acnes* supported by an active Th1 immune response to the bacterium during the process of granulomatous inflammation, as shown in Fig 4.

The fact that *P*. *acnes* reactivity was not detected in some patient samples, even within sarcoid granulomas of the CS-group, requires further investigation. One possible explanation is that other bacteria, such as *Propionibacterium granulosum* and *Mycobacterium tuberculosis*, cause granuloma formation in some sarcoid samples. The anti-*P*. *acnes* antibody used in the present study was specific to *P*. *acnes* and did not cross-react with these bacteria. The mycobacterial etiology of sarcoidosis has long been suspected in many Western countries, even though bacterial culture from sarcoid lesions and IHC detection of *M*. *tuberculosis* in sarcoid granulomas has been unsuccessful. In this study, we examined all samples with a monoclonal antibody specific to all species of mycobacteria as a control, and found no positive reaction in any samples by IHC with the antibody, which reacts with lipoarabinomannan and detects mycobacteria in tuberculous granulomas of formalin-fixed paraffin-embedded tissue sections [15]. Ishige et al., on the other hand, detected many *P*. *granulosum* genomes in 3 of 15 Japanese sarcoidosis patients [17], yet none of the biopsy samples of the 3 patients with *P*. *granulosum* contained *P*. *acnes*-specific sequences. The findings from other studies using real-time PCR also suggested this possibility [28]. It is also possible that detection of *P*. *acnes* in the granulomas of some CS samples failed because the granuloma cells, which have a more powerful intracellular digestion ability than macrophages, destroyed the *P*. *acnes* antigens [29]. Thus, as the granuloma matured, the *P*. *acnes* antigen could have been totally eradicated from the granuloma cells. According to this possibility, the lack of *P*. *acnes* detection in granuloma cells by IHC does not necessarily rule out sarcoidosis as the cause of the granuloma.

There are several limitations to the present study. First, this was a retrospective study, and the sample size was relatively small. Thus, further studies are needed to generalize the present findings. Second, lower frequency of granulomas in the large tissue samples obtained by surgery or autopsy seemed to be due to persistent inflammation with prominent fibrosis in these samples obtained from end-stage heart failure patients. Third, a higher frequency of granulomas in the small EMB tissue samples may be due to selection bias when we collected the EMB samples. Fourth, subjects of the myocarditis group included only lymphocytic cell type excluding eosinophilic and giant cell types in the present study as results of accurately distinguishing three major cell types of myocarditis by H-E staining. Finally, *P*. *acnes* detection using real-time PCR could not be performed because of the poor quality of the autopsy samples (due to long-term fixation) and the small size of EMB samples. Detection of *P*. *acnes* DNA by in situ hybridization, as described in our previous study [30], was not performed in the present study because the feasibility of the detection method is lower than that of IHC for routine pathologic diagnosis.

There are also limitations for IHC detection of *P*. *acnes* in tissue sections obtained by myocardial biopsy as a diagnostic or clinical tool. IHC results for each sample were defined as positive when at least one signal was detected within any of the granuloma or inflammatory cells. The *P*. *acnes*-positive signals, especially those in inflammatory cells, may not have been detected in the original sections, but only in the deep-cut sections of the biopsy samples. Therefore, we may need to examine both the original and deep-cut sections by IHC as well as HE staining in routine pathologic diagnostic processes. Another limitation was the high percentage of samples excluded from this study due to non-specific background staining. The non-specific staining was mainly caused by strong signals of perinuclear lipofuscin pigments in the myocardial cells in some samples, whereas in other samples these pigments did not produce non-specific reactions. This unfavorable phenomenon seems to have been caused by inappropriate tissue preparation including long-term fixation with formalin and was not observed in recent biopsy samples that were fixed with Carnoy solution.

## Conclusions

The present study implicated *P*. *acnes* in the pathogenesis and diagnosis of CS. Notably, frequent detection of *P*. *acnes* in both granuloma and inflammatory cells in myocardial biopsy samples from CS patients suggests that IHC with the anti-*P*. *acnes* monoclonal antibody can be an additional diagnostic method to the present limited use of EMB in the differential diagnosis of CS from non-sarcoidosis myocarditis and other cardiomyopathies.

## Supporting information

S1 File Clinical and histologic data used for analysis. (XLSX)
